# Prevalence and characterization of carbapenem‐resistant bacteria in water bodies in the Los Angeles–Southern California area

**DOI:** 10.1002/mbo3.692

**Published:** 2018-07-10

**Authors:** Dana E. Harmon, Osvaldo A. Miranda, Ashley McCarley, Michelle Eshaghian, Natasha Carlson, Cristian Ruiz

**Affiliations:** ^1^ Department of Biology California State University Northridge Northridge California

**Keywords:** *Aeromonas*, carbapenem, carbapenemase, carbapenem‐resistant, *Cupriavidus*, *Enterobacter*, *Pseudomonas*, *Stenotrophomonas*

## Abstract

Carbapenems are β‐lactam antibiotics used in healthcare settings as last resort drugs to treat infections caused by antibiotic‐resistant bacteria. Carbapenem‐resistant bacteria are increasingly being isolated from healthcare facilities; however, little is known about their distribution or prevalence in the environment, especially in the United States, where their distribution in water environments from the West Coast has not been studied before. The aim of this study was to determine the prevalence of carbapenem‐resistant bacteria and carbapenemase genes in water bodies from the Los Angeles area (California, USA). All samples that were analyzed contained carbapenem‐resistant bacteria with a frequency of between 0.1 and 324 carbapenem‐resistant cfu per 100 mls of water. We identified 76 carbapenem‐resistant or ‐intermediate isolates, most of which were also resistant to noncarbapenem antibiotics, as different strains of *Enterobacter asburiae*,* Aeromonas veronii*,* Cupriavidus gilardii*,* Pseudomonas,* and *Stenotrophomonas* species. Of them, 52 isolates were carbapenemase‐producers. Furthermore, PCR and sequence analysis to identify the carbapenemase gene of these carbapenemase‐producing isolates revealed that all *Enterobacter asburiae* isolates had a *bla* _IMI__‐2_ gene 100% identical to the reference sequence, and all *Stenotrophomonas maltophlia* isolates had a *bla*_L1_ gene 83%–99% identical to the reference *bla*_L1_. Our findings indicate that water environments in Southern California are an important reservoir of bacteria‐resistant to carbapenems and other antibiotics, including bacteria carrying intrinsic and acquired carbapenemase genes.

## 1 INTRODUCTION

Carbapenems (ertapenem, imipenem, meropenem and doripenem) are broad‐spectrum β‐lactam antibiotics. Unlike other β‐lactams such as penicillins and cephalosporins, carbapenems are resistant to hydrolysis by β‐lactamases and extended spectrum β‐lactamases (Martin & Kaye, 2004; Papp‐Wallace, Endimiani, Taracila, & Bonomo, 2011; Vardakas, Tansarli, Rafailidis, & Falagas, 2012). The use of carbapenems is generally restricted to hospitals and other healthcare settings, where they are used as last resort drugs to treat serious infections caused by antibiotic‐resistant bacteria (Bradley et al., 1999; Nordmann, Dortet, & Poirel, 2012; Papp‐Wallace et al., 2011; Paterson, 2000, 2002; Paterson & Bonomo, 2005; Torres, Villegas, & Quinn, 2007).

Carbapenem‐resistant bacteria represent a major challenge to public health. These bacteria are primarily considered as nosocomial pathogens (Bratu, Landman, et al., 2005; Bratu, Mooty, et al., 2005; Centers for Disease Control and Prevention, 2013a), and their isolation in healthcare settings is on the rise (Centers for Disease Control and Prevention, 2018; Correa et al., 2012; Cuzon et al., 2011; Guh et al., 2015; Gupta, Limbago, Patel, & Kallen, 2011; Kallen, Hidron, Patel, & Srinivasan, 2010; Khuntayaporn, Montakantikul, Mootsikapun, Thamlikitkul, & Chomnawang, 2012; Prabaker & Weinstein, 2011; Queenan et al., 2012; Rhomberg & Jones, 2009; Rizek et al., 2014; Rodríguez‐Martínez, Poirel, & Nordmann, 2009; van Duijn, Dautzenberg, & Oostdijk, 2011; Viehman, Nguyen, & Doi, 2014). For example, carbapenem‐resistant Enterobacteriaceae have been designated as an urgent threat by the CDC (Centers for Disease Control and Prevention, 2013a, 2013b) and are associated with very high mortality rates (Papp‐Wallace et al., 2011; Paterson, 2000; van Duin, Kaye, Neuner, & Bonomo, 2013). Likewise, *Acinetobacter baumannii* and *Pseudomonas aeruginosa* strains that are resistant to multiple antibiotics, including carbapenems, have been designated by the CDC as serious threats, and are often untreatable (Centers for Disease Control and Prevention, 2013a, 2013b). *Stenotrophomonas maltophilia*, another hard‐to‐treat emerging pathogen that causes pneumonia and blood infections among other diseases (Brooke, 2012), is usually resistant to most antibiotics, including carbapenems (Brooke, 2012; Yang et al., 2014).

A variety of mechanisms can contribute to carbapenem resistance. These include decreased outer membrane permeability (Livermore, Mushtaq, & Warner, 2005; Rizek et al., 2014; Shin et al., 2012; Sho et al., 2013; Warner et al., 2013), overexpression of efflux pumps or chromosomal β‐lactamases (Papp‐Wallace et al., 2011; Rodríguez‐Martínez et al., 2009; Warner et al., 2013), and production of carbapenemases, which are enzymes that degrade carbapenems and other β‐lactams (Marsik & Nambiar, 2011; Queenan & Bush, 2007). Carbapenemase production is especially worrisome because of the strong activity of these enzymes against carbapenems, and the fact that carbapenemase genes are frequently found in genetic mobile elements such as plasmids, which favors their spread (Mathers et al., 2011; Walsh, 2010).

Despite their public health importance and increased incidence in healthcare facilities (Guh et al., 2015; Gupta et al., 2011; Papp‐Wallace et al., 2011; Prabaker & Weinstein, 2011; Rhomberg & Jones, 2009; van Duijn et al., 2011), knowledge about carbapenem‐resistant bacteria and genes in the environment is very limited, especially in the United States. Most efforts to detect these bacteria have focused on healthcare (Conlan et al., 2014; Doi & Paterson, 2015; Guh et al., 2015; Gupta et al., 2011) or immediately related settings such as hospital wastewater (Chagas, Seki, da Silva, & Asensi, 2011; Nasri et al., 2017; White et al., 2016). However, recent findings in Europe, Africa and Asia have revealed carbapenem‐resistant bacteria and genes in freshwater and other environmental samples (Di, Jang, Unno, & Hur, 2017; Girlich, Poirel, & Nordmann, 2010; Henriques et al., 2012; Isozumi et al., 2012; Poirel et al., 2012; Potron, Poirel, Bussy, & Nordmann, 2011; Tacão, Correia, & Henriques, 2015; Zurfluh, Hachler, Nuesch‐Inderbinen, & Stephan, 2013).

In the United States, carbapenem‐resistant bacteria were isolated from 7 out of 16 rivers from the Midwest sampled between 1999 and 2001 (Ash, Mauck, & Morgan, 2002; Aubron, Poirel, Ash, & Nordmann, 2005). To this date, this study remains the only specific analysis about the distribution and characteristics of carbapenem‐resistant bacteria in water environments not directly related to healthcare facilities in the United States. Given the importance of carbapenem‐resistant bacteria, further studies on other areas of the United States and on different types of aquatic environments, are needed to gain a better understanding of the environmental distribution and molecular mechanisms of carbapenem‐resistant bacteria in the United States. To contribute to address this gap in knowledge, we report here the first study about the distribution and characteristics of carbapenem‐resistant bacteria and carbapenemase genes in aquatic environments on the West Coast of the United States, as well as the first study about these bacteria and genes in ponds and lakes in the United States. All samples analyzed contained carbapenem‐resistant bacteria — most of which were also resistant to other antibiotics — which we identified as *Enterobacter*,* Aeromonas, Cupriavidus, Pseudomonas* or *Stenotrophomonas* species. Many of the carbapenem‐resistant isolates further characterized carried a carbapenemase gene. These findings suggest that carbapenem‐resistant bacteria and carbapenemase genes are widely distributed on diverse water environments on the West Coast of the United States.

## 2 MATERIALS AND METHODS

### 2.1 Sample collection and isolation of carbapenem‐resistant bacteria

**Figure 1:**
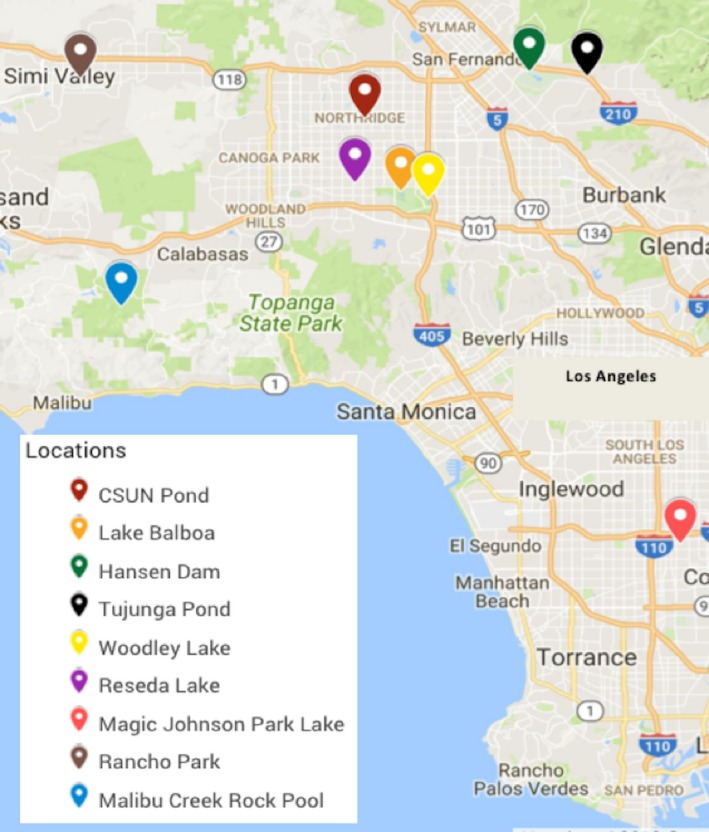
Map of the location of the ponds and lakes from the Los Angeles‐Southern California area sampled in this study

We collected 10 different water samples from ponds and lakes in the Los Angeles (California) area between June of 2016 and March of 2017. The location (Figure 1) and characteristics of the sampling sites are summarized in Table 1. Four liters of surface‐level water were collected in sterile bottles and immediately transported to the laboratory. The total count of gram‐negative bacteria was determined by direct plating 100 μl of water (and also by spot plating 10 μl of a 10^0^ to 10^–4^ dilution bank of each sample in sterile water) on MacConkey agar (Fisher Scientific, Hampton, NH) plates, followed by incubation in aerobic conditions for 24 hr at 37°C, and colony counting. The count of carbapenem‐resistant gram‐negatives was determined by the same procedure except for using MacConkey agar plates supplemented with 4 μg/ml of meropenem (Ark Pharm, Inc., Arlington Heights, IL), which is the meropenem minimum inhibitory concentration (MIC) clinical breakpoint for Enterobacteriaceae according to the Clinical and Laboratory Standards Institute (CLSI) (Clinical and Laboratory Standards Institute, 2017). Meropenem was the second carbapenem approved for medical use in the United States, and has stronger activity than imipenem against most gram‐negatives — the main target in our studies — such as Enterobacteriaceae (Papp‐Wallace et al., 2011). In addition, we concentrated the bacteria present in 2 L of water sample by filtration, using 0.45 μm filters (Merck Millipore, Billerica, MA), and placed the filters onto MacConkey‐meropenem plates for incubation as described above.

**Table 1:** Summary of the origin, total gram‐negative and carbapenem‐resistant gram‐negative bacterial counts obtained in this study

Sample	Date	Location (Type)	GPS Location	Total bacteria (cfu/100 ml)	Carbapenem‐resistant Bacteria (cfu/100 ml)
W1	6/7/2016	CSUN Duck Pond (artificial pond^a^)	34.2367024, ‐118.5261293	4.2 × 10^5^	150.0
W2	8/2/2016	CSUN Duck Pond (artificial pond^a^)	34.2367024, ‐118.5261293	1.2 × 10^5^	10.0
W3	8/17/2016	Lake Balboa (reclaimed water from DCTWRP^b^)	34.182312, ‐118.495627	2.4 × 10^4^	22.5
W4	9/29/2016	Hansen Dam (flood control reservoir)	34.271505, ‐118.388383	8.3 × 10^4^	42.4
W5	10/5/2016	Tujunga Ponds Wildlife Sanctuary (spring water^c^)	34.268050, ‐118.340026	8.8 × 10^5^	16.0
W6	10/7/2016	Woodley Wildlife Lake (reclaimed water from DCTWRP^b^)	34.177256, ‐118.472841	4.0 × 10^4^	324.0
W7	10/11/2016	Reseda Park Lake (artificial lake with potable water^d^)	34.188714, ‐118.534383	1.0 × 10^4^	0.1
W8	1/29/2017	Magic Johnson Park lake (Potable water^d^)	33.919458, ‐118.261776	1.2 × 10^5^	11.0
W9	1/17/2017	Rancho Simi Community Park Duck Pond (Potable water^d^)	34.266453,‐118.764119	3.9 × 10^4^	36.8
W10	3/1/2017	Malibu Creek Rock Pool (natural pool^e^)	34.096555, ‐118.729879	4.9 × 10^4^	9.2

a *Note*.We obtained two water samples from this artificial pond, one before (June of 2016) and one after (August 2016) it was cleaned and the water–pumping system fixed. This artificial pond uses circulation of potable water.

b Lake Balboa and Woodley Wildlife Lake are filled with reclaimed water from the Tillman Water Reclamation Plant (DCTWRP). Lake balboa is a recreational lake, and Woodley Wildlife lake is a wild wetland habitat with many species of birds.

c The water from the Tujunga Ponds Wildlife Sanctuary is spring water from the Tujunga Canyon delivered to the pond via a small stream.

d Reseda Park Lake, Magic Johnson Park lake, and Rancho Simi Community Park Duck Pond use circulation of potable water. Reseda park lake is an asphalt‐lined urban lake.

e Malibu Creek Rock Pool is a natural pool filled with rain run‐off.

Up to 50 meropenem‐resistant colonies per sample, choosing different colony morphologies whenever possible, were patched the next day in Mueller‐Hinton (Fisher Scientific) agar plates supplemented with meropenem at 4 μg/ml, which is the CLSI meropenem MIC clinical breakpoint for Enterobacteriaceae, and 16 μg/ml, which is the CLSI meropenem MIC clinical breakpoint for other non‐Enterobacteriaceae gram‐negatives (Clinical and Laboratory Standards Institute, 2017), to confirm their resistance to meropenem. Over 90% of the colonies patched were confirmed as meropenem‐resistant and grew at both 4 and 16 μg/ml of meropenem. For each water sample, eight to twelve different carbapenem‐resistant isolates were restreaked on Mueller–Hinton‐meropenem‐16 μg/ml plates and incubated as described above to obtain isolated colonies. One colony per isolate was used to inoculate Mueller–Hinton broth supplemented with meropenem‐16 μg/ml. These cultures were incubated in aerobic conditions with 200 rpm agitation for 18–24 hr at 37°C. A portion of each overnight culture was saved with 20% (v/v) glycerol at −80°C for long‐term storage, and the remainder was washed and used for PCR analysis. Cells were washed twice by centrifugation for 1 min at 13,000 rpm (16,200*g*), supernatant removal, and resuspension in DNA grade water (Fisher Scientific). Washed cells were then stored at −20°C until their use as PCR template for amplification of 16S rDNA or carbapenemase genes.

### 2.2 Identification of carbapenem‐resistant bacteria by 16S rDNA sequencing and oxidase test

The 16S rDNA genes from the 76 selected isolates were amplified using reagents and Dreamtaq polymerase purchased from Thermo Fisher Scientific (Canoga Park, CA), and using the previously described primers 8F and U1492R (Eden, Schmidt, Blakemore, & Pace, 1991), which were purchased from IDT (Coralville, IA). The PCR mixture (50 μl) contained DNA grade water, colorless DreamTaq Buffer, 0.2 mM dNTPs, 1.25 DreamTaq polymerase units, 0.5 μM of each primer, and 5 μl of isolate template (washed cells) prepared as described above. Washed *E. coli* BW25113 cells were used as template positive control, and DNA grade water was used as the nontemplate control. The amplification reaction was performed in a Simpliamp thermal cycler (Applied Biosystems/Thermo Fisher Scientific), using the following program: one cycle at 95°C for 2 min, 35 cycles of 95°C for 30 s, 55°C for 30 s, and 72°C for 90 s, with a final cycle of 72°C for 7.5 min and 4°C for infinite. PCR products were then visualized by DNA electrophoresis before sequencing them at Laragen Inc. (Culver city, CA), and analyzing the resulting sequences by BLAST (Altschul et al., 1997).

Because several species of the genera *Pseudomonas* and *Stenotrophomonas* are closely related and are difficult to distinguish based only on their 16S rDNA sequences, isolates in which their 16S rDNA closely matched both genera were further identified using the oxidase test. This test detects the production of the cytochrome C oxidase enzyme, and is positive for *Pseudomonas* and negative for *Stenotrophomonas* (Bergey & Holt, 1994). The oxidase test was performed using the Becton Dickinson BBL DrySlide Oxidase reagent (Sparks, MD). Briefly, strains were grown at 37°C, overnight on Mueller–Hinton agar plates. A plastic pipette tip was used to transfer a large clump of cells to the DrySlide. Isolates that turned blue within 10 s were scored as positives for the oxidase test. Lab strains of *Pseudomonas stutzeri* and *E. coli* BW25113 were used as our positive and negative controls, respectively.

### 2.3 Determination of the antibiotic susceptibility profile of carbapenem‐resistant isolates

**Figure 2:**
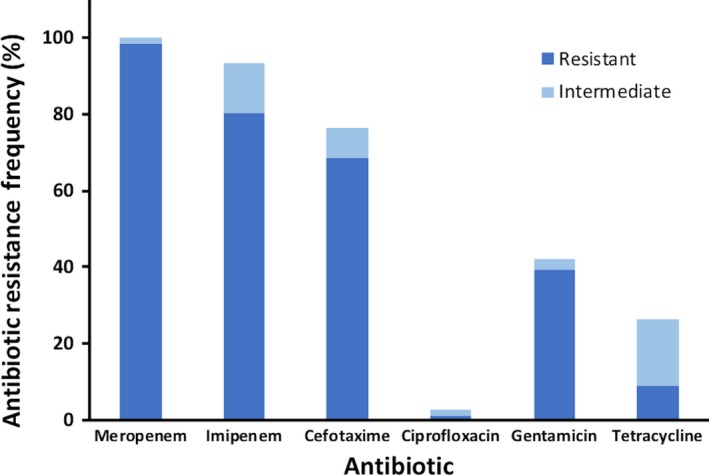
Antibiotic resistance frequency of the water isolates characterized in this study for carbapenem (meropenem and imipenem) and non‐carbapenem (cefotaxime, ciprofloxacin, gentamicin and tetracycline) antibiotics. For each antibiotic tested, the percentage of resistant isolates is shown in dark blue, and the percentage of intermediate isolates is shown in light blue

Determination of the antibiotic susceptibility profile for carbapenems and other antibiotics (Table 2; Table 3; and Figure 2) for each selected isolate was performed using the disk diffusion method as described by CLSI (Clinical and Laboratory Standards Institute, 2017), using cells grown 16–18 hr at 37°C on Mueller‐Hinton agar plates, and using the reference strain *E. coli* ATCC 25922 as a quality control (Clinical and Laboratory Standards Institute, 2017). All antibiotic disks (meropenem 10 μg, imipenem 10 μg, cefotaxime 30 μg, ciprofloxacin 5 μg, gentamicin 10 μg, and tetracycline 30 μg) were purchased from Becton Dickinson (Franklin Lakes, NJ). We used the CLSI zone diameter breakpoint values (Clinical and Laboratory Standards Institute, 2017) to determine whether our isolates were resistant, intermediate, or sensitive to the different antibiotics tested. For taxa in which the CLSI zone diameter breakpoint values were not available, we used the Enterobacteriaceae values.

**Table 2:** Number and characteristics of carbapenem‐resistant isolates identified from water samples described in Table 1

Species	Sample of Origin	Number of isolates	Number of CP^a^ isolates	Carbapenemase gene^a^	Antibiotic Resistance (number of isolates)^b^
*Enterobacter asburiae*	W6	7	7	*bla*_IMI‐2_	MP (7), IM (7)
*Aeromonas veronii*	W7	2	0	N/A	MP (1), IM (1), TE (1)
*Cupriavidus gilardii*	W2, W8	2	0	N/A	MP (2), GE (2)
*Pseudomonas alcaligenes*	W1, W4, W5	5	0	N/A	MP (5), IM (1), CF (4)
*Pseudomonas cedrina*	W9	1	0	N/A	MP (1), IM (1), CF (1)
*Pseudomonas geniculata*	W8	1	0	N/A	MP (1), GE (1)
*Pseudomonas otitidis*	W3–5	9	0	N/A	MP (9), IM (2)
*Pseudomonas stutzeri*	W3	1	0	N/A	MP (1), IM (1), CI (1)
*Stenotrophomonas maltophilia*	W1–4, W8–10	45	45	*bla*_L1_	MP (45), IM (45), CF (44), GE (25), TE (6)
*Stenotrophomonas pavanii*	W5	3	0	N/A	MP (3), IM (3), CF (3), GE (2)
Total		76	52	52	MP (75), IM (61), CF (52), CI (1), GE (30), TE (7)

a *Note*.CP = carbapenemase‐producing as determined by the CarbaNP test. CarbaNP‐positive isolates were further tested by PCR and sequencing to identify their carbapenemase gene, whereas CarbaNP‐negative isolates were not further tested and are shown as N/A in the carbapenemase gene column.

b In parentheses, the number of isolates that were resistant (intermediate isolates are not included) to meropenem (MP), imipenem (IM), cefotaxime (CF), ciprofloxacin (CI), gentamicin (GE), and tetracycline (TE). The detailed antibiotic susceptibility profile, CarbaNP result and carbapenemase gene detected for each individual isolate are provided in Table 3.

**Table 3:** Carbapenem‐resistant isolates identified and characterized in this study

Closest species identified by BLAST using 16S rDNA gene^a^	Isolate#	Inhibition zone (diameter in mm)^b^						Carba NP^c^	Carbape nemase gene (%identity^d^)
		MP	IM	CF	CI	GE	TE		
*Aeromonas veronii*	W7‐1	23	20	40	39	27	25	–	N/A
*Aeromonas veronii*	W7‐2	5	0	31	34	22	11	–	N/A
*Cupriavidus gilardii*	W2‐2	1	20	40	36	0	28	–	N/A
*Cupriavidus gilardii*	W2‐5	2	20	40	36	0	29	–	N/A
*Enterobacter asburiae*	W6‐1	0	0	32	39	27	28	+	*bla*_IMI‐2_ (100%)
*Enterobacter asburiae*	W6‐2	0	0	34	39	27	27	+	*bla*_IMI‐2_ (100%)
*Enterobacter asburiae*	W6‐3	0	0	31	39	25	26	+	*bla*_IMI‐2_ (100%)
*Enterobacter asburiae*	W6‐4	0	0	32	34	25	25	+	*bla*_IMI‐2_ (100%)
*Enterobacter asburiae*	W6‐5	0	0	36	41	26	29	+	*bla*_IMI‐2_ (100%)
*Enterobacter asburiae*	W6‐7	0	0	35	39	27	28	+	*bla*_IMI‐2_ (100%)
*Enterobacter asburiae*	W6‐8	0	0	37	36	28	28	+	*bla*_IMI‐2_ (100%)
*Pseudomonas alcaligenes*	W1‐4	18	26	29	39	24	22	–	N/A
*Pseudomonas alcaligenes*	W4‐5	11	26	15	47	29	27	–	N/A
*Pseudomonas alcaligenes*	W5‐5	8	15	10	38	25	24	–	N/A
*Pseudomonas alcaligenes*	W5‐7	17	24	9	34	25	21	–	N/A
*Pseudomonas alcaligenes*	W5‐8	14	25	12	40	26	20	–	N/A
*Pseudomonas cedrina*	W9‐8	11	13	16	27	33	33	–	N/A
*Pseudomonas geniculata*	W8‐10	0	21	43	37	0	31	–	N/A
*Pseudomonas otitidis*	W3‐5	11	20	26	33	26	25	–	N/A
*Pseudomonas otitidis*	W4‐1	15	22	27	41	29	23	–	N/A
*Pseudomonas otitidis*	W4‐2	14	24	29	39	32	21	–	N/A
*Pseudomonas otitidis*	W4‐3	1	21	27	41	31	25	–	N/A
*Pseudomonas otitidis*	W4‐6	10	18	24	39	28	22	–	N/A
*Pseudomonas otitidis*	W4‐7	10	21	27	39	30	23	–	N/A
*Pseudomonas otitidis*	W4‐8	10	21	25	37	29	21	–	N/A
*Pseudomonas otitidis*	W5‐3	14	22	25	30	24	20	–	N/A
*Pseudomonas otitidis*	W5‐4	0	18	21	30	22	15	–	N/A
*Pseudomonas stutzeri*	W3‐4	11	19	22	14	23	25	–	N/A
*Stenotrophomonas maltophilia*	W1‐2	0	0	0	19	0	8	+	*bla*_L1_ (99%)
*Stenotrophomonas maltophilia*	W1‐3	0	0	8	26	21	20	+	*bla*_L1_ (84%)
*Stenotrophomonas maltophilia*	W1‐5	0	0	10	26	24	22	+	*bla*_L1_ (83%)
*Stenotrophomonas maltophilia*	W1‐6	0	0	0	26	22	21	+	*bla*_L1_ (90%)
*Stenotrophomonas maltophilia*	W2‐1	0	0	13	27	12	14	+	*bla*_L1_ (92%)
*Stenotrophomonas maltophilia*	W2‐3	0	0	24	26	19	19	+	*bla*_L1_ (84%)
*Stenotrophomonas maltophilia*	W2‐4	0	0	11	25	11	15	+	*bla*_L1_ (89%)
*Stenotrophomonas maltophilia*	W2‐6	2	0	12	29	11	13	+	*bla*_L1_ (89%)
*Stenotrophomonas maltophilia*	W2‐7	0	0	12	25	11	12	+	*bla*_L1_ (89%)
*Stenotrophomonas maltophilia*	W2‐8	0	0	13	37	12	13	+	*bla*_L1_ (89%)
*Stenotrophomonas maltophilia*	W3‐1	0	0	11	25	11	11	+	*bla*_L1_ (89%)
*Stenotrophomonas maltophilia*	W3‐2	0	0	11	22	5	11	+	*bla*_L1_ (92%)
*Stenotrophomonas maltophilia*	W3‐6	0	0	0	22	12	12	+	*bla*_L1_ (99%)
*Stenotrophomonas maltophilia*	W3‐7	0	0	12	26	11	15	+	*bla*_L1_ (89%)
*Stenotrophomonas maltophilia*	W3‐8	0	0	12	28	14	14	+	*bla*_L1_ (89%)
*Stenotrophomonas maltophilia*	W4‐4	0	0	0	24	16	13	+	*bla*_L1_ (93%)
*Stenotrophomonas maltophilia*	W8‐1	0	0	12	37	21	20	+	*bla*_L1_ (89%)
*Stenotrophomonas maltophilia*	W8‐2	0	0	0	26	7	14	+	*bla*_L1_ (94%)
*Stenotrophomonas maltophilia*	W8‐3	0	0	9	31	23	22	+	*bla*_L1_ (84%)
*Stenotrophomonas maltophilia*	W8‐4	0	0	0	24	19	11	+	*bla*_L1_ (94%)
*Stenotrophomonas maltophilia*	W8‐5	0	0	0	24	17	11	+	*bla*_L1_ (94%)
*Stenotrophomonas maltophilia*	W8‐7	0	0	0	37	15	20	+	*bla*_L1_ (94%)
*Stenotrophomonas maltophilia*	W8‐8	0	0	0	37	18	20	+	*bla*_L1_ (92%)
*Stenotrophomonas maltophilia*	W8‐9	0	0	13	37	25	20	+	*bla*_L1_ (88%)
*Stenotrophomonas maltophilia*	W8‐11	0	0	13	37	20	19	+	*bla*_L1_ (89%)
*Stenotrophomonas maltophilia*	W8‐12	0	0	13	37	27	21	+	*bla*_L1_ (89%)
*Stenotrophomonas maltophilia*	W9‐1	0	0	11	32	11	20	+	*bla*_L1_ (84%)
*Stenotrophomonas maltophilia*	W9‐2	13	0	11	29	12	18	+	*bla*_L1_ (83%)
*Stenotrophomonas maltophilia*	W9‐3	0	0	9	37	25	23	+	*bla*_L1_ (89%)
*Stenotrophomonas maltophilia*	W9‐4	0	0	9	30	0	16	+	*bla*_L1_ (88%)
*Stenotrophomonas maltophilia*	W9‐5	0	0	11	32	27	21	+	*bla*_L1_ (84%)
*Stenotrophomonas maltophilia*	W9‐6	0	0	0	28	11	13	+	*bla*_L1_ (94%)
*Stenotrophomonas maltophilia*	W9‐7	0	0	16	29	11	15	+	*bla*_L1_ (92%)
*Stenotrophomonas maltophilia*	W9‐12	0	0	10	34	26	24	+	*bla*_L1_ (83%)
*Stenotrophomonas maltophilia*	W10‐1	0	0	0	28	10	17	+	*bla*_L1_ (88%)
*Stenotrophomonas maltophilia*	W10‐2	0	0	0	27	8	16	+	*bla*_L1_ (89%)
*Stenotrophomonas maltophilia*	W10‐3	0	0	0	28	11	15	+	*bla*_L1_ (89%)
*Stenotrophomonas maltophilia*	W10‐5	0	0	0	29	6	14	+	*bla*_L1_ (89%)
*Stenotrophomonas maltophilia*	W10‐6	0	0	0	27	10	15	+	*bla*_L1_ (89%)
*Stenotrophomonas maltophilia*	W10‐7	0	0	0	27	9	15	+	*bla*_L1_ (89%)
*Stenotrophomonas maltophilia*	W10‐8	0	0	0	27	16	14	+	*bla*_L1_ (88%)
*Stenotrophomonas maltophilia*	W10‐9	0	0	0	28	4	14	+	*bla*_L1_ (89%)
*Stenotrophomonas maltophilia*	W10‐10	0	0	0	27	15	11	+	*bla*_L1_ (85%)
*Stenotrophomonas maltophilia*	W10‐11	0	0	10	28	0	15	+	*bla*_L1_ (84%)
*Stenotrophomonas maltophilia*	W10‐12	0	0	13	28	0	14	+	*bla*_L1_ (92%)
*Stenotrophomonas pavanii*	W5‐1	0	0	12	32	13	18	–	N/A
*Stenotrophomonas pavanii*	W5‐2	0	0	11	27	9	17	–	N/A
*Stenotrophomonas pavanii*	W5‐6	0	0	10	28	0	17	–	N/A

a *Note*.For each isolate, we obtained their 16S rDNA sequence and used BLAST (Altschul et al., 1997) to determine the closest known strain. In all cases, the DNA identity between our isolate and the top BLAST known strain hit was ≥98% (≥99% for most isolates).

b MP: meropenem; IM: imipenem; CF: cefotaxime; CI: ciprofloxacin; GE: gentamicin; TE: tetracycline. To determine whether our isolates were Resistant (highlighted in red), Intermediate (highlighted in yellow) or Sensitive (no highlight) to the antibiotics tested, we used the CLSI zone diameter clinical breakpoint values (Clinical and Laboratory Standards Institute, 2017). For taxa in which the CLSI zone diameter breakpoint values were not available, we used the Enterobacteriaceae values.

c All CarbaNP‐positive isolates (carbapenemase‐producing isolates) were positive when the test was performed measuring the hydrolysis of both meropenem and imipenem.

d Only carbapenemase‐producing isolates (CarbaNP‐positive isolates) were tested by PCR to identify their potential carbapenemases. The rest of isolates were not tested because they were CarbaNP‐negative and are shown as N/A. “%identity” indicates % DNA identity (shown in parenthesis) between the reference *bla*_IMI‐2_ or *bla*_L1_ gene and the isolate *bla*_IMI‐2_ or *bla*_L1_ sequence obtained for that isolate.

### 2.4 Identification of carbapenemase‐producing isolates by the CarbaNP assay

We used the CarbaNP assay (Dortet, Poirel, & Nordmann, 2012a, 2012b; Nordmann, Poirel, & Dortet, 2012) to identify which carbapenem‐resistant isolates produced carbapenemases. The assay was performed as described by CLSI (Clinical and Laboratory Standards Institute, 2017) using 6 mg/ml of either meropenem or imipenem, and using colonies from isolates grown overnight at 37°C on Mueller–Hinton agar without (to detect for noninducible carbapenemases) or with (to detect for carbapenem‐inducible carbapenemases) meropenem at 4 or 16 μg/ml. Isolates that hydrolyzed meropenem and/or imipenem, and thus turned yellow at 37°C within 2 hr, but did not turn yellow in the absence of meropenem or imipenem, were considered positive for carbapenemase production. Carbapenemase production was considered carbapenem‐inducible when the CarbaNP test was positive only when using cells grown in Mueller–Hinton‐meropenem plates.

### 2.5 PCR, sequencing and phylogenetic analysis of carbapenemases

PCR amplification and sequencing of carbapenemases from CarbaNP‐positive isolates were performed as described in section 2.2, with the following modifications: We used the primers and PCR program described by Henriques et al. (2012) to amplify *bla*_L1._ To amplify *bla*_IMI_, we designed the primers Imi2‐F1 (5’‐CAA GTA GAA TAG CCA TCT TGT TTA G) and Imi2‐R1 (5’‐AGG TTA TCA ATT GCG ATT CTT G), which amplify 853 out of 870 bp of the *bla*_IMI‐1_ (U50278) and *bla*_IMI‐2_ (DQ173429) genes, and performed the PCR step using a Tm of 55°C and an extension time of 1 min. For each PCR, washed cells of strains carrying each *bla* gene were used as positive control, *E. coli* BW25113 was used as negative control, and DNA grade water was used as nontemplate control. We used Geneious R11 software to perform multiple sequence alignments (MUSCLE alignment tool) of the *bla*_IMI‐2_ sequences obtained and the *bla*_IMI‐2_ reference sequence (DQ173429), and of the *bla*_L1_ sequences obtained and the *bla*_L1_ reference sequence (NG_047502), as well as to build a *bla*_L1_ phylogenetic tree based on the Jukes–Cantor genetic distance model and the Neighbor‐Joining method.

### 2.6 Nucleotide accession numbers

All 16S rDNA, *bla*_IMI‐2_, and *bla*_L1_ sequences obtained in this study have been deposited in GenBank (https://www.ncbi.nlm.nih.gov/genbank/) under the following accession numbers: MG905248–MG905292, MG905294–MG905307, MG905309–MG905321, and MH200608–MH200614 for 16SrDNA sequences, MH203307–MH203307 for *bla*_IMI‐2_, and MG882588–MG882609 and MG882611–MG882634 for *bla*_L1_ sequences_._

## 3 RESULTS

### 3.1 Distribution, frequency and identification of carbapenem‐resistant gram‐negative bacteria in water bodies in the Los Angeles area

We analyzed 10 different water samples from ponds and lakes in the Los Angeles area (California, United States) and found that all of them contained carbapenem (meropenem)‐resistant gram‐negative bacteria. The frequency of gram‐negative meropenem‐resistant bacteria was between 0.1 and 324 meropenem‐resistant cfu per 100 mls of water, which represented between 0.002% and 0.8% of the total gram‐negative bacteria found in these water samples (Table 1). The two samples with the highest count of meropenem‐resistant bacteria per 100 ml were W1 and W6. Location W1 (CSUN Duck pond) is an artificial pond with a large number of animals (ducks, geese and turtles) which uses circulated potable water. However, the circulation system was not functioning at the time of sampling (the same location sampled after cleaning the pond and fixing the circulation system had more than a 10‐fold decrease in the number of carbapenem‐resistant bacteria, whereas the total number of gram‐negatives was only reduced by less than 4‐fold). Location W2 (Woodley Wildlife Lake) has an extensive population of animals, particularly birds, and uses reclaimed water from a nearby water treatment facility. The rest of the water samples, which include natural ponds as well as ponds and lakes that use circulation of potable or reclaimed water had comparable total numbers of carbapenem‐resistant bacteria, except for Reseda Park Lake, an asphalt‐lined lake that had a very low number of carbapenem‐resistant bacteria.

We selected a total of 76 meropenem‐resistant/intermediate isolates (about 8 per sample) for further identification and characterization. Using their 16S rDNA sequence (and oxidase test results when necessary), we preliminarily identified them as 7 *Enterobacter asburiae*, 2 *Aeromonas veronii,* 2 *Cupriavidus gilardii*, 5 *Pseudomonas alcaligenes*, 1 *Pseudomonas cedrina*, 1 *Pseudomonas geniculata*, 9 *Pseudomonas otitidis*, 1 *Pseudomonas stutzeri*, 45 *Stenotrophomonas maltophilia*, and 3 *Stenotrophomonas pavanii* strains (Table 2). Among the isolates selected for identification and characterization, the genera *Stenotrophomonas* and *Pseudomonas* were both the most abundant and widespread; that is, 48 selected isolates collected from nine different water samples were *Stenotrophomonas,* and 17 isolates collected from 6 different water samples were *Pseudomonas*.

### 3.2 Characterization of the antibiotic susceptibility profile of carbapenem‐resistant isolates

We characterized the antibiotic susceptibility profile of the 76 identified carbapenem‐resistant or ‐intermediate isolates using disk diffusion experiments with 2 carbapenems (meropenem and imipenem) and 4 non‐carbapenem (cefotaxime, ciprofloxacin, gentamicin, and tetracycline) antibiotics (Table 2; Table 3; and Figure 2). Overall, 99% of our isolates (all except for one intermediate *A. veronii*) were resistant to meropenem, as expected by our use of meropenem as the selective agent to obtain these isolates. Most isolates (all except for four *P. alcaligenes* and one *P. otitidis*) were also resistant (80%) or intermediate (13%) to imipenem. For the non‐carbapenem β‐lactam cefotaxime, most isolates were also resistant (68%) or intermediate (8%). These were all *Stenotrophomonas* and about two thirds of all *Pseudomonas*. In contrast, resistance to non‐β‐lactam antibiotics was much lower (Table 2; Table 3; and Figure 2). For ciprofloxacin, 97% of the isolates (all isolates except for one *S. maltophilia* and one *P. stutzeri*) were sensitive. For gentamicin, 39% of the isolates were resistant and 3% were intermediate. Gentamicin‐resistant/intermediate isolates were *C. gilardii*,* P. geniculata*, and two thirds of all *Stenotrophomonas*. For tetracycline, 9% and 17% of the isolates were resistant or intermediate, respectively. These were mostly *S. maltophilia* and one *A. veronii* isolate (Table 2; Table 3; and Figure 2). Overall, these findings highlight the importance of different aquatic environments in the Los Angeles‐Southern California area as reservoirs of bacteria that are resistant to carbapenems and other antibiotics.

### 3.3 Identification of carbapenemases from carbapenem‐resistant isolates

We next used the CarbaNP test to identify which carbapenem‐resistant or ‐intermediate isolates produce carbapenemases. We found that 52 out of the 76 isolates studied were positive for carbapenemase production when tested using meropenem and/or imipenem (Table 2; Table 3). CarbaNP‐positive isolates included 7 *E. asburiae* and all 45 *S. maltophilia*. In all these isolates, their carbapenemases were inducible.

**Figure 3:**
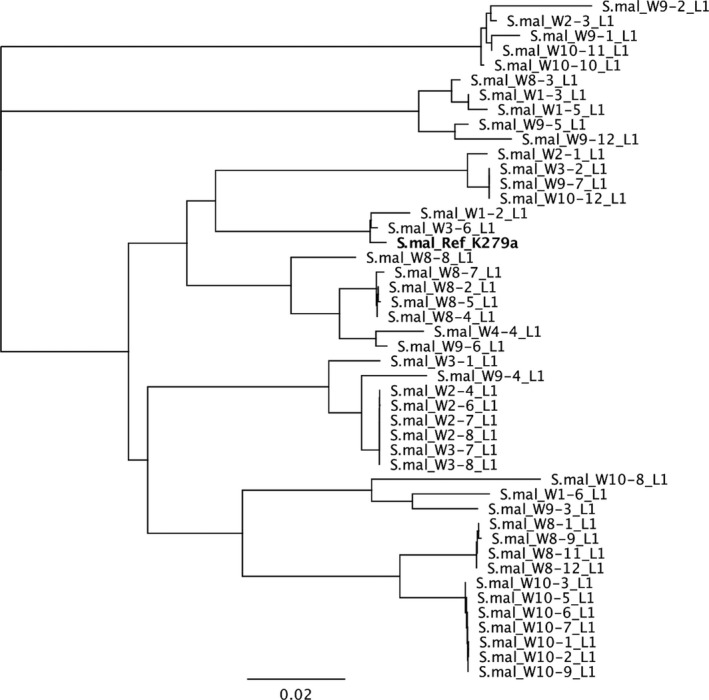
Phylogenetic tree showing relatedness between the reference *bla*_L1_ gene sequence and the L1 carbapenemases gene sequences obtained in this study. The tree was constructed using the Neighbor‐Joining method. The scale bar at the bottom represents the number of nucleotide substitutions per site. In bold the *S. maltophilia* strain K279a *bla*_L1_ reference sequence (NG_047502). Abbreviations: S.mal: *Stenotrophomonas maltophilia*

We then used PCR and sequencing to identify the carbapenemase genes present in the 52 CarbaNP‐positive isolates (Table 2; Table 3; and Figure 3). All seven *E. asburiae* isolates had a *bla*_IMI‐2_ gene 100% identical to the *bla*_IMI‐2_ reference sequence (DQ173429). All 45 carbapenemase‐producing *S. maltophilia* isolates had the *bla*_L1_ gene. Analysis of the *bla*_L1_ genes identified revealed that environmental L1 carbapenemases are very diverse. All *bla*_L1_ DNA sequences obtained for our isolates had between 83% to 99% identity to the reference *S. maltophilia bla*_L1_ gene (NG_047502) — a variability similar to that found in *S. maltophilia* clinical isolates (Avison, Higgins, von Heldreich, Bennett, & Walsh, 2001).

## 4 DISCUSSION

Carbapenem resistance is one of the major threats to public health worldwide (Centers for Disease Control and Prevention, 2013a, 2013b; Guh et al., 2015; Gupta et al., 2011; Papp‐Wallace et al., 2011; Prabaker & Weinstein, 2011; Rhomberg & Jones, 2009; van Duijn et al., 2011). Despite the significance of carbapenem‐resistant bacteria, there is little information about these bacteria outside healthcare or immediately related facilities (Chagas et al., 2011; Conlan et al., 2014; Doi & Paterson, 2015; Guh et al., 2015; Gupta et al., 2011; Nasri et al., 2017; White et al., 2016), especially in the United States. To this date, the only study about these bacteria in aquatic environments in the United States focused on rivers from the Midwest sampled between 1999 and 2001 (Ash et al., 2002; Aubron et al., 2005), a time when carbapenem use and the spread of carbapenem‐resistant bacteria in clinical settings were much lower than they are today (Centers for Disease Control and Prevention, 2018; Pakyz, MacDougall, Oinonen, & Polk, 2008).

Our study is the first one in more than a decade to investigate the distribution, frequency, antibiotic susceptibility profile, and carbapenemase genes of carbapenem‐resistant bacteria in aquatic environments in the United States. This study is also the first one to study carbapenem‐resistant bacteria in environmental water bodies on the West Coast of the United States, as well as the first one to study the distribution, frequency and characteristics of carbapenem‐resistant bacteria in non‐riverine water environments such as ponds and lakes in the United States. We found that gram‐negative bacteria resistant to carbapenems and other antibiotics are widespread in water bodies in the Los Angeles‐Southern California area. We could detect and isolate carbapenem‐resistant bacteria from all ponds and lakes tested with a frequency ranging from 0.002% to 0.8% of the total gram‐negative bacteria present in the samples analyzed. Although this frequency cannot be directly compared to the results found for rivers in the midwestern United States sampled between 1999 and 2001 because imipenem‐resistant isolates were identified by screening isolates first identified as ampicillin resistant (Ash et al., 2002; Aubron et al., 2005), the frequency of carbapenem‐resistant bacteria found in our study is similar to that found Tacão et al. (2015) in Portuguese rivers.

Characterization of a total of 76 isolates from these samples showed that carbapenem‐resistant or ‐intermediate bacteria in ponds and lakes from the Los Angeles area are quite diverse, and include different species preliminarily identified as *Enterobacter asburiae*,* Aeromonas veronii, Cupriavidus gilardii*,* Pseudomonas alcaligenes*,* Pseudomonas cedrina*,* Pseudomonas geniculata*,* Pseudomonas otitidis*,* Pseudomonas stutzeri*,* Stenotrophomonas maltophilia*, and *Stenotrophomonas pavanii*. These results have some similarities and differences with previous studies. The most abundant carbapenem‐resistant bacterium among our isolates (found in most of our samples) was *S. maltophilia*, which is common in aquatic environments and intrinsically resistant to carbapenems (Brooke, 2012). However, we also found *S. pavanii* in the Tujunga pond (a natural pond filled with spring water). *S. pavanii* is a bacterium previously found in plants (Ramos et al., 2011) and in bird feces (Kenzaka & Tani, 2018), which to our knowledge has not been found before in aquatic environments.

The second most abundant and widespread (present in most of our samples) group of carbapenem‐resistant bacteria we found was *Pseudomonas*. Interestingly, carbapenem‐resistant *Pseudomonas* isolates (*P. geniculata* and P. *otitidis* among other *Pseudomonas*, but not *P. cedrina* or *P. stutzeri*) were also found to be abundant in Portuguese rivers (Tacão et al., 2015), but were not found in the midwestern United States rivers (Aubron et al., 2005). In contrast with the results for *Pseudomonas*,* Enterobacter asburiae* represented the most abundant and widespread (they were found in 4 different rivers) carbapenem‐resistant isolate found in rivers from the U.S. Midwest (Aubron et al., 2005), but was only found in one sample both in the study of Portuguese rivers (Tacão et al., 2015) as well as in our study (Woodley Wildlife lake).

The other carbapenem‐resistant or ‐intermediate isolates that we found are *Aeromonas veronii* and *Cupriavidus gilardii*. *Aeromonas*, including *A. veronii*, are common water inhabitants and are often intrinsically resistant to carbapenems (Aubron et al., 2005; Lupo, Coyne, & Berendonk, 2012; Tacão et al., 2015). In contrast, this is the first time that carbapenem‐resistant *C. gilardii* isolates — which we identified in a location (W2) that uses recirculated potable water — have been reported outside of clinical settings (Karafin et al., 2010; Kobayashi et al., 2016).

To further characterize the 76 selected carbapenem‐resistant or ‐intermediate isolates, we used disk diffusion antibiotic susceptibility experiments with carbapenem (meropenem and imipenem) and noncarbapenem antibiotics (cefotaxime, ciprofloxacin, gentamicin, and tetracycline). The antibiotics we chose have different cellular targets, entry routes, and resistance mechanisms. Therefore, even if not all these antibiotics are clinically used to treat all of the genera that we identified, they provide very important information about the potential antibiotic resistance mechanisms found in these isolates. For example, strains generally very resistant to most or all antibiotics suggest an important role of general antibiotic resistance mechanism such as decreased outer membrane permeability and/or increased efflux by multidrug efflux pumps, in addition to more specific mechanisms. Strains that are only resistant to one antibiotic or class of antibiotics suggest that such resistance is likely to be predominantly caused by specific mechanisms such as target mutations or antibiotic degrading enzymes such as carbapenemases. In general, resistance to carbapenems, β‐lactams (cefotaxime), aminoglycosides (gentamicin) and tetracyclines (tetracycline) was widespread among our isolates, whereas resistance to fluoroquinolones (ciprofloxacin) was very rare among them.

Resistance to carbapenems can occur by different mechanisms such as production of carbapenemases, overexpression of efflux pumps, and decreased outer membrane permeability (Livermore et al., 2005; Marsik & Nambiar, 2011; Papp‐Wallace et al., 2011; Queenan & Bush, 2007; Rizek et al., 2014; Rodríguez‐Martínez et al., 2009; Shin et al., 2012; Sho et al., 2013; Warner et al., 2013). Production of carbapenemases seems to be a major contributing mechanism for carbapenem‐resistance in most of our isolates because 52 of the 76 isolates characterized were carbapenemase‐producers. The carbapenemase gene of these carbapenemase‐producing isolates was identified by PCR and sequencing as *bla*_IMI‐2_ 100% identical to the reference sequence for all *Enterobacter asburiae* isolates and as *bla*_L1_ for all *Stenotrophomonas maltophilia* isolates. The *bla*_L1_ carbapenemase genes identified showed varying diversity (83%–99% DNA identity) compared to the *bla*_L1_ reference sequence. The presence of the L1 carbapenemase in *Stenotrophomonas maltophilia* is well documented in both clinical and environmental isolates and is a major contributor to its untreatability (Brooke, 2012; Tacão et al., 2015; Youenou et al., 2015).

Of greater concern is the identification of seven *E. asburiae* isolates carrying the *bla*_IMI‐2_ carbapenemase gene. This gene is an inducible plasmid‐encoded carbapenemase gene that was first identified in carbapenem‐resistant *E. asburiae* isolates from four different U.S. Midwest rivers (Aubron et al., 2005), and was later found in an *Enterobacter cloacae* isolate recovered from river sediment in Spain in 2017 (Piedra‐Carrasco et al., 2017). *bla*_IMI‐2_ has also recently been found in clinical isolates of *E. asburiae* (Czech Republic) (Rotova et al., 2017), *E. cloacae* (China) (Yu, Du, Zhou, Chen, & Li, 2006), *Escherichia coli* (Spain and China) (Rojo‐Bezares, Martin, Lopez, Torres, & Saenz, 2012; Zhang et al., 2017), and *Klebsiella variicola* (United Kingdom) (Hopkins, Findlay, Doumith, Mather, & Meunier, 2017). In both environmental and clinical isolates, *bla*_IMI‐2_ was usually found in transposable elements located in transferable plasmids (Aubron et al., 2005; Hopkins et al., 2017; Piedra‐Carrasco et al., 2017; Rojo‐Bezares et al., 2012; Rotova et al., 2017; Yu et al., 2006; Zhang et al., 2017); however, one *E. asburiae* clinical isolate carrying the *bla*_IMI‐2_ gene in its chromosome (the transposable element was not characterized) was identified in South Africa in 2015 (Gqunta et al., 2015). Our results show that this acquired carbapenemase gene is also spread outside of clinical and river environments, which may be related to the presence of *bla*_IMI‐2_ in transposable elements located in transferable plasmids (Aubron et al., 2005; Hopkins et al., 2017; Piedra‐Carrasco et al., 2017; Rojo‐Bezares et al., 2012; Rotova et al., 2017; Yu et al., 2006; Zhang et al., 2017). Further, surveillance is necessary to better characterize the role of freshwater environments as a source of IMI‐2‐producing *E. asburiae*, which can be both an opportunistic pathogen, as well as a reservoir of this transferable carbapenemase.

In conclusion, our findings show for the first time that freshwater environments in Los Angeles‐Southern California represent an underappreciated reservoir of bacteria resistant to carbapenems and other antibiotics, many of which carry intrinsic or acquired carbapenemase genes.

## ACKNOWLEDGMENTS

This work was supported by the California State University Northridge start‐up funds to C. Ruiz, and was also partially supported by the National Institutes of Health BUILD PODER 5RL5GM118975‐03 and the California State University CSUPERB New Investigator grants to C. Ruiz. We thank William Jackson for his help in the analysis of *bla*_L1_ sequences.

## CONFLICT OF INTEREST

The authors declare that the research was conducted in the absence of any potential sources of conflict of interest.

## DATA ACCESSIBILITY STATEMENT

The authors adhere to all policies on sharing data and materials described in the guidelines for authors.
